# Supplementary material for: Genome Sequencing and Transcriptome Profiling in Twins Discordant for Mayer-Rokitansky-Küster-Hauser Syndrome
Source: J Clin Med. 2022 Sep 23;11(19):5598. doi: 10.3390/jcm11195598 (PMC9573672; doi:10.3390/jcm11195598)
Supplement: Supplementary file 1 [file jcm-11-05598-s001.zip › jcm-1861930-supplementary.pdf]

Journal of
Clinical Medicine

MDPI

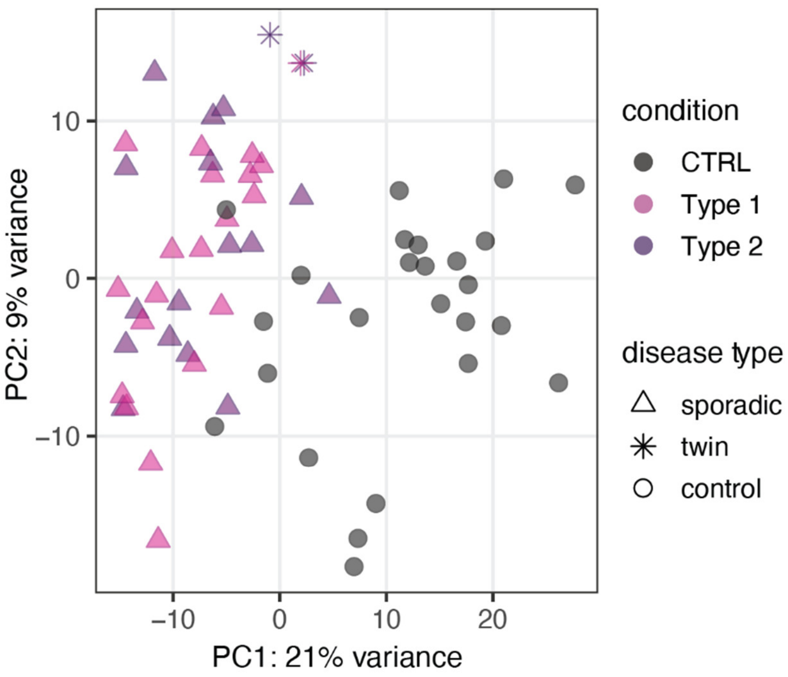

Supplementary Figure S1. Both MRKH twin and sporadic samples separated from control tissue in unaffected individuals.

Principal component analysis of endometrial gene expression profiles for three affected MRKH twins, 35 sporadic MRKH patients (19 type 1 and 16 type 2) as well as 25 unaffected controls. Axis percentages indicate variance contribution.

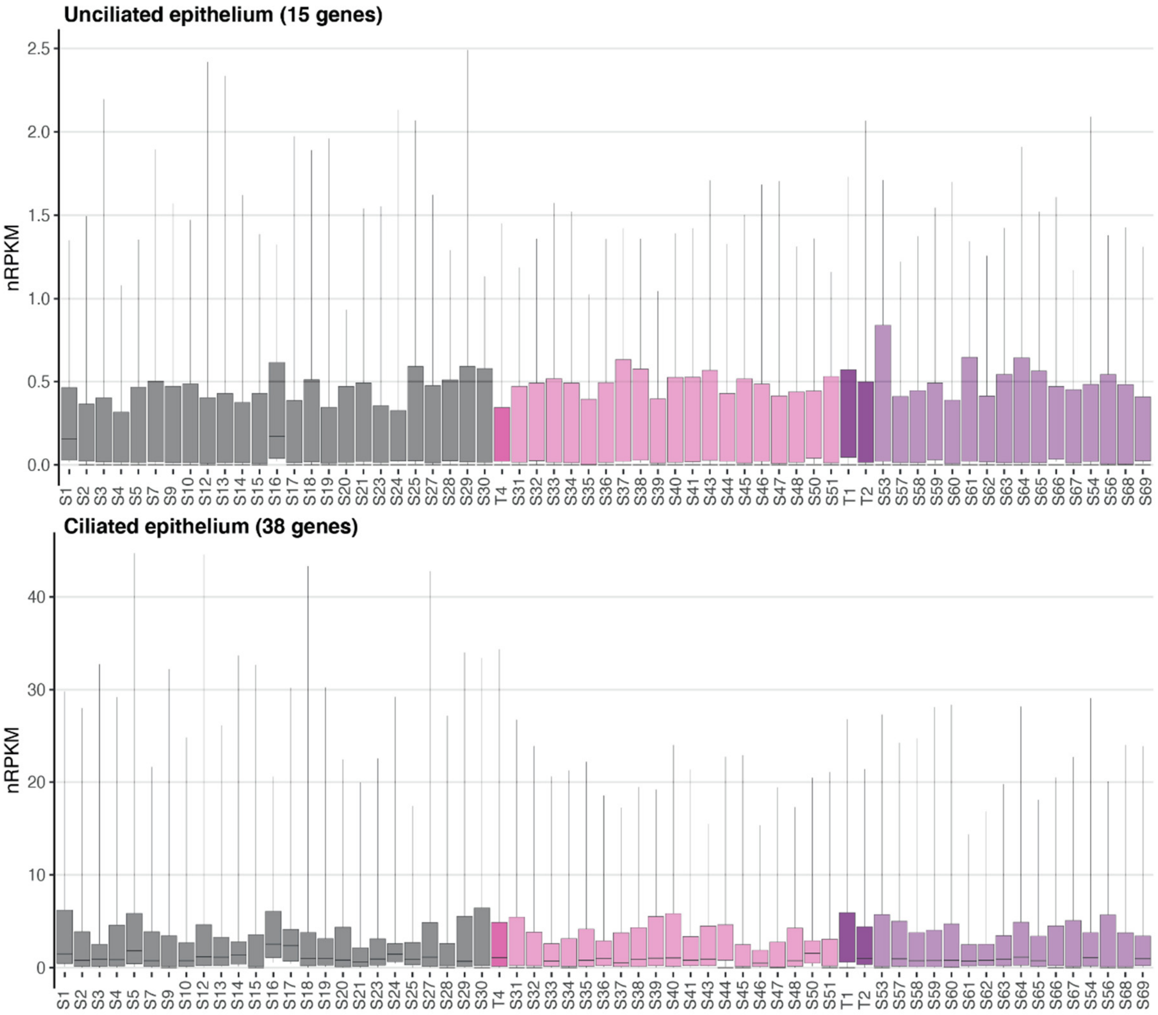

Supplementary Figure S2. Gene expression of cell type-specific markers is similar between MRKH twins, sporadic cases, and unaffected controls.

Cell type-specific gene expression per sample for unciliated and ciliated epithelium. Boxplots show geometric mean as well as 10th, 25th, 75th, and 90th quantile of expression values for all genes classified based on single-cell data of the human endometrium [20]. Number of genes per cell type in brackets.

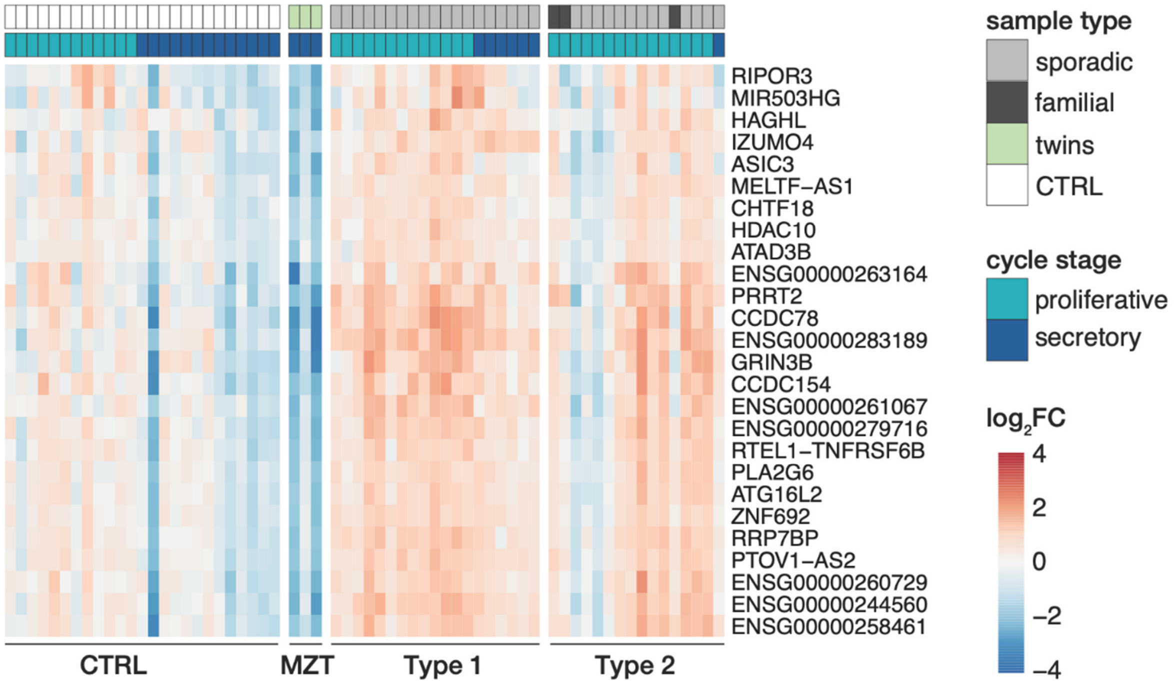

Supplementary Figure S3. Subgroup of genes showing opposing expression changes between MRKH twins and sporadic cases.

Expression profiles (*log*$_2$ expression change relative to Ctrl group) of 26 DEGs that show opposing expression changes between twins and sporadic cases (subgroup of DEGs from Figure 3C) across all samples. Rows hierarchically clustered by Euclidian distance and *ward.D2* method. Cycle information (proliferative or secretory) and patient type (monozygotic twin, sporadic, or control) on top.
